# Homing instinct

**DOI:** 10.1017/S1478951525100552

**Published:** 2025-09-12

**Authors:** Tarek Zieneldien

**Affiliations:** Johns Hopkins University School of Medicine, Baltimore, MD, USA

I was born beside the mango tree
Grew tall, reaching for the baby green mangos
I craved the sour skin that hid the innocence behind the seed
I was always close but never close enough –
Especially once entire oceans stood between us

So, when I received the news
My homing instinct was too strong to resist
The only thing worse than dying
Was dying in a land that was not my own
I craved resting my head on my grandmother’s mango tree
Then using fallen branches to knock down baby green mangos –
A diorama of my childhood

Before explaining the next steps
Take me back
I want to hear them in my native tongue
I want to understand
And process the gravity of the situation
Beneath the shade of the mango tree

